# Redesigning medicine: integrating sex and gender for better health outcomes

**DOI:** 10.1007/s00210-025-04722-7

**Published:** 2025-10-22

**Authors:** Luisa S. Rajcsanyi, Maren A. Jochimsen, Andrea Kindler-Röhrborn, Anke Hinney

**Affiliations:** 1 Section of Molecular Genetics in Mental Disorders, LVR-University Hospital Essen, University of Duisburg-Essen, Essen, Germany; 2 Institute of Sex- and Gender-Sensitive Medicine, University Hospital Essen, Essen, Germany; 3 Center for Translational and Behavioural Neuroscience, University Hospital Essen, Essen, Germany; 4 Essen College of Gender Research, University of Duisburg-Essen, Essen, Germany

**Keywords:** Sex-sensitive medicine, Gender, Personalised medicine, Biological determinants

## Abstract

Both the biological sex and the socio-cultural gender play a central role in medical care, influencing diagnosis, disease progression, and treatment decisions. Despite well-established and substantial differences between men and women, sex-specific differentiation in guidelines, and dosing protocols remain limited. This gap in care has deep historical roots: for decades, women were systematically excluded from clinical trials resulting in a pronounced sex bias in medical research. Although recent efforts have been made to address these inequalities, women are still under-represented in many clinical trials. However, recent inclusive research has highlighted the relevance of a sex- and gender-sensitive medicine for women and men. It is becoming increasingly acknowledged that diseases that preponderately affect women, such as breast cancer or depression, can also manifest in men. This recognition has prompted the development of sex- and gender-sensitive diagnostic tools, for example in the assessment of depression. Similarly, in the context of myocardial infarction, it is now well established that the classical signs of a heart attack are predominantly male-specific manifestations, while women experience a range of symptoms that do not fully overlap with the symptoms in males. Here, we will take a closer look at sex- and gender-specific differences in medicine, highlight key historical developments and milestones and discuss prospects for the future sex- and gender sensitive medicine.

## Introduction

Modern medicine has been fundamentally shaped by the perception of the male body as the norm, reinforced by the enduring assumption that women are merely a smaller version of men. Historically, political regulations have restricted female participation in clinical trials, causing biomedical studies to predominantly employ male cells, animals, and participants (Madla et al. 2021; Mauvais-Jarvis et al. 2020; Recio-Barbero and Pérez-Fernandez 2019). 1

Yet, despite a growing awareness of sex- and gender-specific differences in disease prevalence, clinical presentation, and therapeutic response among all stakeholders, translation into clinical practice remains limited. Sex-specific guidelines are currently only available for a small number of diseases (Regitz-Zagrosek 2023b). Nonetheless, already acquired knowledge and further emerging evidence of sex and gender differences highlight the impact of an inclusive healthcare system with benefits for both, women and men.

Here, we outline historical developments that have shaped sex- and gender-based disparities in medicine, explore biological and social determinants on health, and present clinical examples highlighting the urgent need for inclusive and sex- and gender-sensitive approaches in research and clinical practice.

## Medicine’s male default and its historical origins

Contemporary medicine is largely based on a male default due to regulatory actions and a historical lack of scientific surveillance (see Table 1). A key driver for these developments was the thalidomide tragedy. In the 1960 s, reports emerged that thalidomide, a drug prescribed for nausea during pregnancy, caused severe birth defects. Worldwide, more than 20,000 children were affected, prompting thalidomide’s ban in most countries (Kim and Scialli 2011; Recio-Barbero and Pérez-Fernandez 2019). Following this tragedy, the United States (US) Food and Drug Administration (FDA) implemented a policy prohibiting the inclusion of women of childbearing potential in early phases of clinical trials (Food and Drug Administration 1977). Although intended as a protective measure for foetal health, this decision had profound and lasting consequences on women’s health and the perception of sex and gender as relevant research variables. Still, current clinical guidelines as well as treatment and dosing protocols are largely based on data derived from male study populations (Madla et al. 2021).

**Table 1:** Some relevant milestones for the integration of sex and gender in medical research studies

Year	Event	Description
1988	Publication of the *Report of the Public Health Service Task Force on Women’s Health* (Public Health Service Task Force on Women's Health 1985)	• Recognition of a lack of knowledge regarding the health of women • Provided relevant recommendations
1990	Foundation of the *Office of Research on Women’s Health* (*NIH*; Mazure and Jones 2015; White et al. 2021)	• Has initiated policies and funded projects on women’s health and the impact of sex and gender on disease and health
1993	*NIH Revitalization Act* (National Institutes of Health 1993)	• Mandated NIH-funded studies to include women
1994	*NIH Guidelines on the Inclusion of Women and Minorities as Subjects in Clinical Research* (Mazure and Jones 2015; White et al. 2021)	• Included guidelines how to analyse and report sex and gender as well as ethnic differences in Phase III clinical trials
2000	First report published regarding effectiveness of prior policies: *Women’s Health: NIH Has Increased Its Efforts to Include Women in Research* (Mazure and Jones 2015)	• Provided evidence that progress in incorporating women in clinical trials has been made • Yet, reported that studies were not systematically analysing data by sex
2001	Report on *Drug Safety: Most Drugs Withdrawn in Recent Years Had Greater Health Risks for Women* (Heinrich 2001)	• Reported that 8 out of 10 prescription drugs approved by the FDA had to be withdrawn since 1997—reason: greater health risks in women
2005	Publication of first trial investigating the use of aspirin to prevent cardiovascular disease in women (Ridker et al. 2005)	• Previous studies only performed in men • Showed that regular aspirin intake (every other day) reduced the risk of stroke in women over 65
2011	Foundation of expert group *Innovation through Gender* by the European Commission (European Commission 2013; White et al. 2021)	• Aimed to provide methods for a sex- and gender-sensitive analysis • Performed case studies to highlight the relevance of sex and gender analyses
2015	*NIH* policy on *Sex as a Biological Variable* (SABV; Mazure and Jones 2015; White et al. 2021)	• Mandated researchers to consider both sexes in vertebrate and human studies • If no sex-specific mechanisms were expected: a reasonable justification needed to be given
2016	*Sex and Gender Equity in Research* (SAGER) guidelines (Heidari et al. 2016)	• Provided recommendations and tools how to report sex and gender in publications • Aimed to improve the reporting of sex and gender in science
2021—2027	*Horizon Europe* (Mazure and Jones 2015; White et al. 2021)	• Funding programme for research in Europe • Aimed to address gender equality by integrating gender dimensions into research and increasing gender balance among research teams • Applied to many scientific disciplines, not restricted to medical sciences

Despite the recognition of a general lack of knowledge about women’s health in the 1980s (Public Health Service Task Force on Women's Health 1985), it was not until the National Institutes of Health (NIH) Revitalization Act of 1993 that regulatory action was taken. This novel policy mandated the inclusion of women in NIH-funded research (National Institutes of Health 1993). However, compliance remained inconsistent as many researchers still favoured male study populations due to assumptions of an increased heterogeneity resulting from the female menstrual cycle (Madla et al. 2021; Mauvais-Jarvis et al. 2020; Mazure and Jones 2015; Mielke and Miller 2021; Recio-Barbero and Pérez-Fernandez 2019). Of note, a meta-analysis of murine studies did not find any evidence for female animals to be more variable than males across various traits. In some cases, male mice even showed a greater heterogeneity (Prendergast et al. 2014). However, numerous drugs still prescribed today were approved without consideration or evaluation of sex-specific differences in efficacy and safety, partly due to this misconception (Madla et al. 2021).

In recent years, regulatory bodies and funding agencies worldwide have increasingly acknowledged the relevance of sex- and gender-sensitive research and medicine (see Table 1). Institutions such as the European Commission have integrated these aspects into their funding programs like the Framework Programme 6 and Horizon Europe (2021–2027; White et al. 2021). Many countries like Canada and the UK demand researchers to consider integration and analyses of both sexes in grant proposals (Mauvais-Jarvis et al. 2020; Recio-Barbero and Pérez-Fernandez 2019; Witt et al. 2024). In fact, a recent study demonstrated the positive effects of regulatory policies and funding calls on the output of sex- and gender-sensitive research. Countries with progressive regulations and targeted funding in this field tend to produce a greater amount of high-quality research. In countries with weaker mandates, editorial policies provided by scientific journals played a compensatory role in helping to sustain research performance (Kim et al. 2024).

Although many studies still fail to include both sexes or report sex-specific findings, the field of sex- and gender-sensitive medicine is progressing, with a growing number of studies considering sex and gender as relevant variables (e.g., Garcia-Sifuentes and Maney 2021; Rechlin et al. 2022). Between 2009 and 2019, the number of studies in neurology and psychiatry examining both women and men increased by 30%. Yet, many of those studies lacked study designs capable of reliably identifying sex-related differences (Rechlin et al. 2022). In general, the future of sex- and gender-sensitive medicine remains uncertain—particularly in the US. Due to ongoing political developments and recent restrictions under the Trump administration, the field is likely to experience a significant regression in the upcoming years. Particularly as Trump has publicly asserted that sex is strictly binary (White House 2025), public datasets have been modified without clear annotations (Freilich and Kesselheim 2025), and publications containing terms such as “gender” or “trans” have been threatened with retraction (Allebeck et al. 2025; Clark and Abbasi 2025; Fender and Hinney 2025; Krieger and Gruskin 2025). This is especially concerning as US-hosted resources, such as PubMed or ClinVar, provide critical datasets and tools for researchers worldwide. Alterations or removal of these datasets without proper documentation could have profound consequences for global research and healthcare. Fortunately, many journals have already expressed their commitment not to comply with unjustified retractions of publications (e.g., Allebeck et al. 2025; Clark and Abbasi 2025; Krieger and Gruskin 2025). Those strong regulations governing research including sex and gender variables, as well as studies investigating the health of women and transgender people is likely to result in the US losing its previous pioneering role and is a threat to the public health and research worldwide (Fender and Hinney 2025). Consequently, it is essential that researchers in other countries feel encouraged to further promote and advance the field of sex- and gender-sensitive medicine.

## Variability shaped by biological sex

Differences by sex are largely determined by the sex chromosomes (X and Y in humans) and the gonadal hormones. An individual with two X chromosomes is biologically considered female, while an individual who carries both, an X and Y chromosome, is considered male. At the genomic level, differences between sex chromosomes arise due to the shorter length of the Y chromosome and because many Y-linked genes either lack homologous counterparts on the X chromosome or have evolved to be functionally distinct. For instance, the *SRY* gene (sex-determining region Y), is essential for testis development. In its absence, as in individuals with an XX karyotype, female differentiation is triggered (Mauvais-Jarvis et al. 2020; Silkaitis and Lemos 2014).

Given the genomic structure of the X chromosome, it is associated with post-transcriptional regulation of gene expression through microRNAs (miRNAs). Accordingly, the X chromosome encompasses approximately 10% of all known miRNAs representing the highest density of miRNAs in comparison to autosomes and the Y chromosome, which only contains four miRNA sequences (Di Palo et al. 2020; Guo et al. 2009). Further, the gene encoding the androgen receptor is located on the X chromosome. In contrast, genes encoding the oestrogen receptors are located on autosomes (Mielke and Miller 2021).

The presence of two X chromosomes can cause gene dosage effects, which is frequently compensated through a random inactivation of one of the X chromosomes in XX cells. Yet, some genes may escape this inactivation, resulting in elevated gene expression levels in females compared to males (Mauvais-Jarvis et al. 2020; Mielke and Miller 2021; Silkaitis and Lemos 2014).

Gonadal hormones such as oestrogen, progesterone, and testosterone are produced by both sexes, albeit in varying levels and with dynamic changes across the lifetime. Oestrogen, with higher levels in females, is primarily synthesised in ovaries, adrenal glands, and adipose tissue. It is essential for the menstrual cycle, reproductive function, and lipid metabolism (Xega and Liu 2024). Female oestrogen levels fluctuate throughout the reproductive years, peak during pregnancy, and decline after menopause (Madla et al. 2021; Mielke and Miller 2021). In males, oestrogen is present at lower levels regulating libido, erectile function, and spermatogenesis. Like oestrogen, progesterone regulates the menstrual cycle, but is also essential for implantation and foetal development. In males, it also contributes to spermatogenesis. Testosterone, the main androgenic hormone, is produced in the testes and adrenal glands. It drives the development of male secondary sexual characteristics and reproductive function (Xega and Liu 2024). Testosterone levels first surge in utero towards the end of the first trimester, remain low throughout childhood, rise during puberty, and subsequently gradually decline with advancing age (Mauvais-Jarvis et al. 2020; Mielke and Miller 2021).

## Sex-based variations in physiology and drug response

Numerous studies have demonstrated that the efficacy and safety of drugs can vary between men and women due to inherent biological differences (Haack et al. 2009; Madla et al. 2021; Regitz-Zagrosek 2023a; Thurmann 2007; Zucker and Prendergast 2023). Despite this, sex-specific dosing and treatment guidelines remain largely absent in clinical practice. As a result, women are often more susceptible to experience adverse drug reactions (ADRs) and overdosing (Regitz-Zagrosek 2023a; Thurmann 2007; Zucker and Prendergast 2023). Accordingly, a 2001 report by the US Government Accountability Office revealed that eight of ten prescription drugs were withdrawn from the market between 1997 and 2000 due to more severe adverse effects in women (Heinrich 2001). Similarly, 31% of drugs evaluated by the FDA between 1994 and 2000 showed significant pharmacokinetic differences between the sexes (Fadiran and Zhang 2015). In fact, women often have longer elimination rates and higher blood concentrations of drugs. For example, among 86 drugs evaluated, 76 had higher pharmacokinetic values in females, many of which were associated with a higher incidence of ADRs in women (Zucker and Prendergast 2020). Consequently, pharmacokinetic and pharmacodynamic processes are impacted by sex. Yet, these differences are frequently overlooked during drug development and evaluation (Madla et al. 2021).

The way in which drugs are absorbed, distributed, metabolised, and eliminated is shaped by physiological differences between men and women (Madla et al. 2021; Zucker and Prendergast 2023). In fact, women generally have a higher body fat percentage, an elevated body mass index (BMI), and an increased gastric pH compared to men. In contrast, men typically have higher total body water, plasma volume, and average organ blood flow (Regitz-Zagrosek 2023a; Madla et al. 2021).

Drug absorption, for example, is affected by longer gastric emptying times in women which is partly attributed to the inhibitory effects of oestrogen and progesterone on intestinal motility (Regitz-Zagrosek 2023a). This results in sex-specific variations in drug uptake. For example, both aspirin and the antihistamine mizolastine are absorbed more rapidly in women. In the case of mizolastine, the average absorption time is about 40 minutes in women, whereas a male’s body takes up to three hours (Mesnil et al. 1998).

Drug metabolism, which primarily takes place in the liver, is often linked to variations in hepatic enzyme expression. *CYP1A2*, for example, is more highly expressed in males, whereas the metabolism of *CYP3A* substrates is faster in females (Hunt et al. 1992; Madla et al. 2021; Oliva et al. 2020; Scandlyn et al. 2008; Schmidt et al. 2001; Xie et al. 2017; Yoon et al. 2021), with clearance rates approximately 20–30% higher (Hunt et al. 2008; Madla et al. 2021).

Renal elimination processes, including glomerular filtration, tubular secretion, and reabsorption differ by sex, with women generally exhibiting lower rates (Madla et al. 2021; Zucker and Prendergast 2023). However, these effects can be substance specific. Thus, clearance of acetaminophen (paracetamol) and heparin is slower in women triggering stronger ADRs (Campbell et al. 1998; Miners et al. 1983; Rubin et al. 2018). Conversely, orally administered aspirin is cleared more rapidly in women and its metabolite, salicylate, shows higher absorption rates in females (Aarons et al. 1989).

Notably, absorption, metabolism, and excretion of drugs are altered during pregnancy and after menopause. Still, pregnant women remain severely underrepresented in clinical trials and medical research (Madla et al. 2021; Recio-Barbero and Pérez-Fernandez 2019). For instance, one study revealed that pregnant women were excluded from SARS-CoV-2 treatment trials, often without valid justification (Taylor et al. 2021)—a concerning issue given that pregnant women with COVID-19 have higher risks of admission to intensive care units (Allotey et al. 2020).

Pharmacodynamic differences emerge when men and women exhibit distinct physiological responses to the same plasma concentration of a drug (Madla et al. 2021). This is particularly evident in the female response to vaccines, where women often require lower doses. One study, for example, reported that women who received half the standard influenza vaccine dose achieved equivalent levels of antibody titres to those in males receiving the full dose (Engler et al. 2008).

Although these pronounced sex-specific differences are well-known, sex-specific dosing recommendations are still lacking (Regitz-Zagrosek 2023a; Zucker and Prendergast 2023). One exception is the sedative zolpidem. It was approved by the FDA in 1992, prior to the NIH Revitalization Act. Post-marketing studies revealed that women had higher concentrations and longer elimination rates of zolpidem. These factors contributed to an impaired driving ability in women following drug administration. Consequently, the FDA mandated that females only needed half the dose men received (Bush 2014; Food and Drug Administration (FDA) 2013; Norman et al. 2017).

## Gender as a social construct in clinical contexts

Alongside the biological sex of individuals, the social gender also affects the overall health. The term “gender” covers roles, behaviours, relationships, and interactions, as well as the identity of individuals that are shaped by their respective societal context. Unlike the biological sex, which is usually described in binary terms, gender is a historically and culturally variable and dynamic category (e.g., Regitz-Zagrosek 2023b; Villa 2019).

Patient behaviour, disease awareness, and the use of the healthcare system have direct impacts on disease manifestation and progression. Lifestyle factors such as diet, smoking, alcohol consumption, physical activity, and the general stress level are closely linked to health outcomes and are often modulated by gender-specific roles and norms (Mauvais-Jarvis et al. 2020). One example is the cutaneous melanoma: men are generally less likely to engage in preventive behaviours such as applying sunscreen or wearing protective clothing, resulting in a higher incidence of melanoma (Adams et al. 2021; Kakish et al. 2025; see Table 2). Of note, in younger individuals (< 50 years of age), females are more often diagnosed with melanoma, while in older ages (> 50 years of age) melanoma is more prevalent in males (Buja et al. 2022). Generally, men working in traditionally “male” sectors, such as construction and road building for example, tend to be particularly at risk. Self-examination is less likely in males and they less frequently consult a physician for suspicious skin lesions contributing to an often-delayed diagnosis and treatment initiation as well as a higher melanoma-related mortality (Adams et al. 2021; Kakish et al. 2025).

**Table 2:** Gender- and sex-specific characteristics of certain medical conditions

Disease	Incidence and prevalence	Differences in symptoms and/or risk factors	Diagnosis, treatment, and outcomes	References
Anorexia nervosa	• Meta-analyses of lifetime prevalence rates reported rates of 4% in females and 0.3% in males • Male:female ratio = 1:13–1:14	• Females: food restriction, purging behaviour or excessive physical activity to reduce weight; want to be thin • Males: behaviour related to gain muscles, such as excessive weight training or use of anabolic steroids; Want to gain muscles, while having low body fat	• Diagnostic criteria have been changed to no longer exclude men (removal of amenorrhoea criterion) • Diagnostic instruments only partially validated in males • Studies on sex- and gender-specific treatment outcomes remain limited: One study found evidence that men with AN had better outcomes than women	van Eeden et al. 2021 Halbeisen et al. 2022; Halbeisen et al. 2024; Hirtz and Hinney 2020
Breast cancer	• Incidence rates in UK: Females: 194.4 per 100,000 person/year males: 1.16 per 100,000 person/year	• Specific risk factors in males: e.g., high oestrogen levels; men with Klinefelter syndrome are particularly at risk	• Males often present with advanced-stage tumours due to limited awareness • Women tend to be diagnosed at around 62 years of age, while males tend to be older with almost 70 years of age • Diagnosis in men may be delayed, as other conditions appear more likely (e.g., gynaecomastia) • Males receive the same treatment as women	Barclay et al. 2024; Bhardwaj et al. 2024; Giordano 2018; Giordano et al. 2004; Siegel et al. 2020
Depression	• Women are twice as likely to be diagnosed with depression than men • Depression-related suicide attempts more frequently in females, but more often lethal in males	• Females: loss of interest, changes in weight and eating behaviour • Males: aggression, substance abuse, risky behaviour	• Diagnostics instruments often do not include male-specific symptoms, but novel instruments have been implemented (GSDS) • Women more likely to seek help • Some studies indicate that women have better treatment outcomes than men when treated with serotonergic antidepressants; pre-menopausal women show better responses than post-menopausal women	Sramek et al. 2016 Friedel et al. 2024; Mauvais-Jarvis et al. 2020; Vonneilich et al. 2025; Yang et al. 2024
Melanoma	• Incidence: < 50 years of age: females more often affected than males > 50 years of age: males more often affected	• Females: melanoma occur more often on extremities, such as arms and legs • Male: melanoma occur more often on the trunk • Men less likely to engage in preventive behaviours such as applying sunscreen or wearing protective clothing • Men often work in “male-specific traditional” jobs such as construction with higher sun exposure	• Studies regarding therapy outcomes still inconclusive • Targeted therapy (BRAF/MEK inhibitors): some studies find better survival in women, others do not find differences • Immunotherapy (anti-PD-1): Some studies report better outcomes in men, others do not find differences	Adams et al. 2021; Botticelli et al. 2017; Buja et al. 2022; Conforti et al. 2018; Kakish et al. 2025; Petersen et al. 2024; Tasdogan et al. 2025; Vellano et al. 2022
Myocardial infarction	• Males more often affected by cardiovascular diseases • Risk in women increases following menopause	• Females: nausea, pain in back and upper abdomen, fatigue • Males: chest pain with left-sided projections in the arm • Yet, overlapping symptoms are possible as well	• Women are admitted later to the hospital • Women receive less often guideline-based therapies • Males more often referred to cardiovascular assessment by cardiologists or general practitioners • Higher mortality rate in women treated by male physicians than those treated by female colleagues	Kuehnemund et al. 2023; Mauvais-Jarvis et al. 2020; Regitz-Zagrosek 2020; Seeland 2023; Shi et al. 2024; Stehli et al. 2021a, b

The impact of gender extends to the healthcare system itself. Studies show that physicians may interact differently with patients based on their perceived gender, affecting diagnosis, and even therapeutic decisions (Mauvais-Jarvis et al. 2020). In pain management, for example, women and their symptoms are more likely to be dismissed or not taken seriously. Concurrently, many women postpone medical consultations due to their competing caregiving responsibilities and concomitant professional commitments (Figueroa and Hiemstra 2024).

Interestingly, the sex of the healthcare providers themselves plays a crucial role for disease progression and outcomes. Studies have shown that when a patient’s sex does not match that of the surgeon, the risk of postoperative complications or even death, increases. This was particularly evident among female patients who were under the care of male surgeons, while outcomes for male patients appear unaffected by the surgeon’s sex (Wallis et al. 2022). Similarly, a study analysing data on myocardial infarction of emergency departments in Florida between 1991 and 2010 reported that female patients treated by female physicians exhibited a better survival rate than those treated by male physicians. Once again, these results were specific to female patients (Greenwood et al. 2018).

The described findings are not only the result of individual decisions, but rather of prevailing gender relations and orders as an interplay of structural and cultural requirements and expectations in the areas of family, work, and leisure. Gender roles and stereotypes not only influence self-image, self-perception and the assessment by others (“doing gender”). They also affect individual health behaviour (“doing health”). “Doing gender” and “doing health” are therefore linked in a broad societal context (Wegrzyn and Jochimsen 2023).

## Clinical manifestations of sex and gender differences

Sex- and gender-based differences have been identified across a wide range of diseases impacting prevalence, incidence, progression, and clinical outcomes (Klein and Flanagan 2016). Recognition and consideration of these differences has led to recent advances in inclusive medicine, improving diagnostic accuracy and tailoring of treatment strategies.

It is already well-established that the prevalence and incidence of diseases vary substantially between men and women. For instance, autoimmune diseases predominantly affect women, while malignant cancers are more frequently diagnosed in men. The effects of biological sex and, for some diseases, factors of gender, play a relevant role. Women, for example, have a stronger innate and adaptive immune system providing enhanced protection against infections and stronger reactions to vaccinations. However, they also have a higher susceptibility to autoimmune diseases and ADRs due to their stronger immune system. In contrast, men have a higher risk of most malignant cancer entities. Exceptions are breast and thyroid cancer, which are more frequently detected in women (Klein and Flanagan 2016; Shi et al. 2024). Notably, male breast cancer is inadequately studied, partly due to the fact that it accounts for only approximately 1% of all breast cancer cases (Siegel et al. 2020; see Table 2). Men are generally diagnosed at a later stage with more advanced stage cancers. On average, men receive a breast cancer diagnosis at the age of 67, while women get diagnosed a few years earlier, at 62 (Giordano et al. 2004). Clinical studies on breast cancer have excluded male participants, similar to the systematic exclusion of women in clinical trials for many other diseases (Bhardwaj et al. 2024; Giordano 2018). Despite well-established risk factors for men, such as elevated oestrogen levels, treatment guidelines for male patients with breast cancer are still extrapolated from female-based studies (Giordano 2018; Madla et al. 2021). Sex-based differences also extend to psychiatric and metabolic disorders. Anorexia nervosa (AN) is predominantly diagnosed in young females (van Eeden et al. 2021; see Table 2). Genome-wide association studies (GWAS) have identified eight genetic loci associated with AN (Watson et al. 2019), and subsequent analyses revealed three overlapping genetic loci linked to both an increased risk for AN and a lower body weight (Hinney et al. 2017). Hereby, one of these loci suggests a sex-specific genetic component (Hinney et al. 2017; Giuranna et al. 2024).

In addition to variations in disease prevalence, disparities are also evident in terms of the disability-adjusted life years (DALYs). A variety of diseases and conditions, including cases of SARS-CoV-2, stroke, and road injuries, have been reported to have elevated DALY rates in the male population. In women, higher DALY rates are shown for conditions such as low back pain, anxiety, depression, and dementia. Interestingly, male-specific DALY rates are associated with premature mortality, while higher DALYs in women are linked to a higher morbidity (Patwardhan et al. 2024).

Two of the most prominent illustrations of the beneficial impacts of sex- and gender-sensitive medicine on the health of both sexes are the myocardial infarction (MI) and depression. For a prolonged period of time, the manifestations of both diseases were characterised by a set of symptoms that were specific to one sex and were accordingly diagnosed leading to a proposed underdiagnosis in the opposing sex (Friedel et al. 2024; Kim et al. 2022; Moller-Leimkuhler & Muhleck 2020; Salokangas et al. 2002; Vogel et al. 2021; see Table 2). Sex- and gender-sensitive research has revealed significant differences, particular in the clinical presentation of patients with both diseases. These findings have the potential to improve the diagnosis and treatment of women with a MI and men with depression.

In general, the prevalence of cardiovascular diseases is higher among males than females (see Table 2). This disparity is primarily attributed to the protective effects of oestrogen in pre-menopausal women (Shi et al. 2024; Lauterbach 2024). As oestrogen levels decline with the onset of menopause, the risk of cardiovascular disease and the associated mortality increases (Shi et al. 2024). MI is arguably the disease for which the differences between the sexes in symptom presentation are most widely recognised in all areas of society. Historically, MI was characterised by the presence of chest pain with a left-sided projection into the arm. However, it should be noted that these symptoms are primarily reported in men. In women, symptoms such as nausea, pain in the upper abdomen, back pain, or fatigue are indicative of MI (Lauterbach 2024; Sandek & Hasenfuß, 2023; Seeland 2023; Stehli et al. 2021a, b). Due to a lack of recognition of these disparities, diagnosis of MI in females was challenging and outcomes were worse. Women were typically admitted later to the hospital due to a lack of awareness across the patients (Diercks et al. 2010; Roswell et al. 2017; Settelmeier et al. 2020; Stehli et al. 2021a, b; Stehli et al. 2021a, b). Once in hospital, women receive guideline-based therapies less frequently than men (Kuehnemund et al. 2023; Stehli et al. 2021a, b). For example, about 75% of women underwent percutaneous coronary intervention, compared to around 85% of men (Kuehnemund et al. 2023). Furthermore, compared to men, women remained on average for about half a day longer in hospital (12.2 vs. 11.7 days) and experienced higher rates of complications. Nearly 8% of women suffered from acute renal failure during their hospital stay, compared to 6.6% of men (Kuehnemund et al. 2023). Even though differences have been recognised for a prolonged period of time, integration into the clinical practice remains insufficient. A German care study (AURORA study) reported on how cardiologists and general practitioners address cardiovascular diseases. Apart from assessing differing symptoms in men and women, male patients were referred for cardiovascular evaluation more frequently than women (Regitz-Zagrosek 2020).

In accordance, sex differences have been identified in prevalence, symptoms, and diagnosis of depression (see Table 2). Women are usually about twice as likely to be affected by depression as men (Friedel et al. 2024; Mauvais-Jarvis et al. 2020; Vonneilich et al. 2025; Williams et al. 2022; Yang et al. 2024). Interestingly, childhood depression is more prevalent in boys than in girls prior to puberty. From around the age of 13, the prevalence of depression in girls increases to exceed that in boys (Frey et al. 2020; Williams et al. 2022). Despite a higher rate of suicide attempts observed in women, the mortality rate following such attempts is higher in men (Miranda-Mendizabal et al. 2019). In Germany, for instance, data from 2022 reveals that 75% of individuals who died by suicide were male (Friedel et al. 2024). The recognition of depression in men is a significant challenge due to the set of symptoms, which deviate considerably from those enumerated in the diagnostic criteria. Diagnosis is primarily based on female symptoms, such as loss of interest, hyperphagia, and weight gain. Conversely, men frequently exhibit symptoms that are not commonly associated with depression, such as aggression, risk-taking behaviour, and substance abuse (Friedel et al. 2024; Mauvais-Jarvis et al. 2020; Vonneilich et al. 2025; Williams et al. 2022; Yang et al. 2024). Therefore, men suffering from depression are potentially underdiagnosed. The diagnosis itself also corroborates this trend. Findings from a prospective study indicated that besides females seeking help more often, general practitioners were less likely to diagnose depression in men, even when clinical depression scores were present and similar to those of females (Bertakis 2009). Commonly used diagnostic tools, such as the Beck depression inventory (BDI), are primarily based on symptom profiles more typical in women. However, when sex-sensitive screening instruments are used (e.g., GSDS (gender-sensitive depression screening); Moller-Leimkuhler & Muhleck 2020) more cases of depression are detected in men (Friedel et al. 2024; Mauvais-Jarvis et al. 2020).

## Shaping the future of sex- and gender-sensitive medicine

Advances in sex- and gender-sensitive medicine have highlighted the significance of integrating and analysing biological sex and social gender as variables in medical research and patient care. Unlike common misconceptions, both women and men benefit, for example through differentiated diagnoses or therapy decisions. Sex- and gender-sensitive medicine is, in fact, a core component of evidence-based and personalised medicine.

Despite a considerable progress in recent years, the field is still in its infancy. Looking ahead, it will be crucial to critically monitor the effects of current political developments in the US on the US (and global) research system and if necessary, take appropriate action. Regardless of political decisions, it will become increasingly important to raise awareness of sex- and gender-based differences at all levels.

Students, medical staff, scientists, and the general public should be educated about relevant factors influencing health outcomes. In particular, students in medicine and the life sciences should be introduced to these concepts early during their training. While elective courses on sex- and gender-sensitive medicine provide a solid foundation, it is essential to integrate these topics into the core curriculum to enable future generations of healthcare providers and researchers to fully recognise, evaluate and embrace sex- and gender-specific differences.

Public awareness campaigns can help to encourage patients to interpret their symptoms and recognise which sex-specific signs may be relevant to them. For example, in the case of an MI, where timely recognition and action can be life-saving (e.g., Cannon et al. 2000; Park et al. 2019), it is essential that patients recognise their symptoms correctly and rapidly. Patients should be educated and motivated to ask their healthcare providers about sex- and gender-specific differences and/or treatment options (Becher and Oertelt-Prigione 2023).

Additionally, scientific journals, funding agencies and regulatory bodies need to establish structural incentives for sex- and gender-sensitive research. Specific funding calls and stringent publication standards that demand transparent reporting of sex and gender of the investigated participants, animals, and even cells, are to be encouraged. Beyond sex and gender, future research, and healthcare must increasingly consider intersecting other dimensions of diversity, such as ethnic origin or socioeconomic status or age, as these factors also impact health outcomes.

To shape and enforce a sex- and gender-sensitive future in medicine, all stakeholders must take an active role in promoting these concepts. The goal is to provide medical care that offers the best possible treatment to all individuals, regardless of their sex and gender. This requires scientists and physicians to take on a pioneering leading role, actively helping to shape the path towards a personalised and inclusive medicine, particularly when facing challenges and opposition.

## Data Availability

All source data for this work (or generated in this study) are available upon reasonable request.

## Footnotes

1 The authors acknowledge that sex and gender exist beyond two categories, which are further not homogenous in themselves. However, this field of research still predominantly relies on a simplified, binary understanding of sex and gender, partly due to the current available data.
